# MeCP2 is a naturally supercharged protein with cell membrane transduction capabilities

**DOI:** 10.1002/pro.5170

**Published:** 2024-09-14

**Authors:** Alexander V. Beribisky, Anna Huber, Victoria Sarne, Andreas Spittler, Nyamdelger Sukhbaatar, Teresa Seipel, Franco Laccone, Hannes Steinkellner

**Affiliations:** ^1^ Institute of Medical Genetics, Center for Pathobiochemistry and Genetics, Medical University of Vienna Vienna Austria; ^2^ Vienna Doctoral School of Pharmaceutical, Nutritional and Sport Sciences (PhaNuSpo), University of Vienna Vienna Austria; ^3^ Core Facility Flow Cytometry & Department of Surgery, Research Laboratories Vienna Austria

**Keywords:** cell penetrating peptides, MeCP2, supercharged protein, TAT fusion proteins

## Abstract

The intrinsically disordered protein MeCP2 is a global transcriptional regulator encoded by the *MECP2* gene. Although the structured domains of MeCP2 have been the subject of multiple studies, its unstructured regions have not been that extensively characterized. In this work, we show that MeCP2 possesses properties akin to those of supercharged proteins. By utilizing its unstructured portions, MeCP2 can successfully transduce across cell membranes and localize to heterochromatic foci in the nuclei, displaying uptake levels a third lower than a MeCP2 construct fused to the cell‐penetrating peptide TAT. MeCP2 uptake can further be enhanced by the addition of compounds that promote endosomal escape following cellular trafficking by means of macropinocytosis. Using a combination of in silico prediction algorithms and live‐cell imaging experiments, we mapped the sequence in MeCP2 responsible for its cellular incorporation, which bears a striking resemblance to TAT itself. Transduced MeCP2 was shown to interact with HDAC3. These findings provide valuable insight into the properties of MeCP2 and may be beneficial for devising future protein‐based treatment strategies.

## 1 INTRODUCTION

Methyl‐CpG‐binding protein 2 (MeCP2) is a known regulator of transcription that possesses both enhancing and silencing capabilities. It imparts these functions via interactions with methylated and unmethylated DNA motifs and chromatin remodeling (Chahrour et al., 2008). This protein is encoded by the X‐linked *MECP2* gene (Lyst & Bird, 2015). Mutations in *MECP2*, which consequently lead to the impairment of MeCP2 activity in the central nervous system (CNS), are the prime cause of Rett syndrome (RTT), a severe neurodevelopmental disease affecting 1 in 10,000 female live births (Amir et al., 1999). It has been demonstrated that re‐establishing cellular MeCP2 presence in the CNS can ameliorate and even reverse some RTT symptoms (Guy et al., 2007). One RTT treatment option, termed protein replacement therapy, has recently shown therapeutic promise (Steinkellner et al., 2022; Zhang, Cattoglio, et al., 2023). This approach involves tethering MeCP2 to a cell‐penetrating peptide (CPP) or a mini‐protein, which can transduce across cell membranes as well as the blood–brain barrier, to deliver MeCP2 into cells. The mechanism of CPP internalization, while not entirely understood, is thought to involve interactions between the basic amino acid side chains of the CPP and the cell surface to initiate the uptake process followed by either endocytosis (with subsequent sampling of the endosomal pathway) or direct translocation (Futaki et al., 2007; Herce et al., 2009; Ruseska & Zimmer, 2020). One such CPP is the 11 amino acid peptide TAT derived from its HIV‐1 namesake protein (Schwarze et al., 1999; Schwarze & Dowdy, 2000), which has exhibited therapeutic potential in treating various neurological disorders (Trazzi et al., 2018; Vyas et al., 2012).

MeCP2 is a basic 53 kDa intrinsically disordered protein (IDP). It is largely devoid of structured elements except two domains: the methyl binding domain (MBD) spanning residues 85–163, and the transcriptional repression domain (TRD), which is located between residues 205 and 310. The MBD has been implicated in binding to methylated and unmethylated DNA sequences (Wakefield et al., 1999) as well as various histones (Ortega‐Alarcon et al., 2024). The TRD was demonstrated to be involved in protein–protein interactions, namely, with histone deacetylase 3 (HDAC3), SIN3A, and TBL1‐related protein 1 (TBLR1) which bring about chromatin remodeling (Kruusvee et al., 2017; Tillotson et al., 2017), as well as with other elements involved in the repression of gene expression (Forlani et al., 2010; Kokura et al., 2001). These functions were shown to be crucial for RTT onset. In fact, a MeCP2 protein variant containing only the MBD and a portion of the TRD, termed the NCoR/SMRT interaction domain (NID) (Lyst et al., 2013), was found to rescue RTT phenotypes in mice as efficiently as its full‐length counterpart (Tillotson et al., 2017).

Although less is known about the disordered portions of MeCP2, the unstructured regions that flank the MBD have been shown to contribute to its overall conformation (Ortega‐Alarcon et al., 2021). Other unstructured parts that harbor RTT‐causing mutations (Buschdorf & Stratling, 2004; Spiga et al., 2019) are known to be implicated in group 2 splicing factor recognition (Buschdorf & Stratling, 2004). In addition, they impart abnormal gel filtration chromatography and SDS–PAGE migration behavior, likely due to the presence of polyproline stretches (Beribisky et al., 2022). Hence, the IDP portion of MeCP2 is likely to be of functional importance.

Supercharged proteins (SPs) are a relatively recently described protein class. While no strict definition criteria for this class have been established, useful parameters outlined in previous studies include the overall charge of the protein and a ratio of the net charge per kDa of molecular weight (CM_W_) above one (Cronican et al., 2011). While some SPs, such as +36 GFP, can be artificially generated (Cronican et al., 2010; Lawrence et al., 2007; Thompson et al., 2012), naturally supercharged human proteins (NSHPs) as well as natural SPs from other natural origins have also been described (Cronican et al., 2011; Zhang et al., 2021). NSHPs are known to possess many unstructured portions (Ma et al., 2020; Wang et al., 2024) which have demonstrated notable durability and resistance to aggregation (Lawrence et al., 2007; Ma et al., 2020). These unstructured parts are deemed important for cellular processes where protein flexibility is vital; for instance, serving as linker elements or being involved in multiple binding partner recruitment. Indeed, a plethora of functions have been attributed to this protein class, such as engaging in DNA/protein interactions, regulating gene expression and signal transduction (Ma et al., 2020; Wang et al., 2024).

Remarkably, similar to CPPs, a number of SPs have also exhibited cell membrane‐penetrating ability. This has been demonstrated with both artificially engineered GFP constructs (Cronican et al., 2010; Thompson et al., 2012) but also with NSHPs such as β‐defensin 3, heparin‐binding EGF‐like growth factor and the basic leucine zipper domain of the transcription factor c‐Jun. In fact, when tethered to the fluorescent reporter protein mCherry, these proteins are transduced across cell membranes with far greater efficiency than their CPP‐mCherry fusion counterparts in vitro (Cronican et al., 2011). In vivo, NSHP‐Cre protein constructs injected into various mouse tissues have been shown to successfully trigger Cre‐induced recombination (Cronican et al., 2011).

In this work, we demonstrated that MeCP2, like other SPs, can penetrate cell membranes. Recently, it was shown that a shortened version of this protein, termed *t*MeCP2 can spontaneously transduce across the membranes of multiple cell lines (Zhang, Cattoglio, et al., 2023). Our work here further expands on these findings. Live‐cell imaging and imaging flow cytometry experiments both demonstrated that MeCP2 utilizes its IDP portion to transduce and localize to various cellular compartments, most strikingly in the nucleus. We also show that this protein samples the endosomal pathway during uptake, specifically macropinocytosis, similar to numerous CPP fusion proteins (Ruseska & Zimmer, 2020). The MeCP2 region implicated in the internalization process was found to be remarkably similar to that of the CPP TAT. Finally, transducing MeCP2 was shown to recruit its known nuclear interaction partner HDAC3. While our work largely focuses on the e2 isoform of MeCP2, some uptake studies were also performed on the RTT‐relevant e1 isoform of this protein (Mnatzakanian et al., 2004; Zachariah et al., 2012), yielding comparable cellular incorporation levels. These findings will further expand our understanding of SPs in general and the properties of MeCP2 in particular. Furthermore, the results here may prove beneficial when exploring potential avenues to treat RTT, other MeCP2‐related disorders (Katz et al., 2016) or any disease for which protein‐based therapy is a viable option.

## 2 RESULTS

### 2.1 Expression and purification of the MeCP2 protein constructs and cell viability assays

**FIGURE 1:**
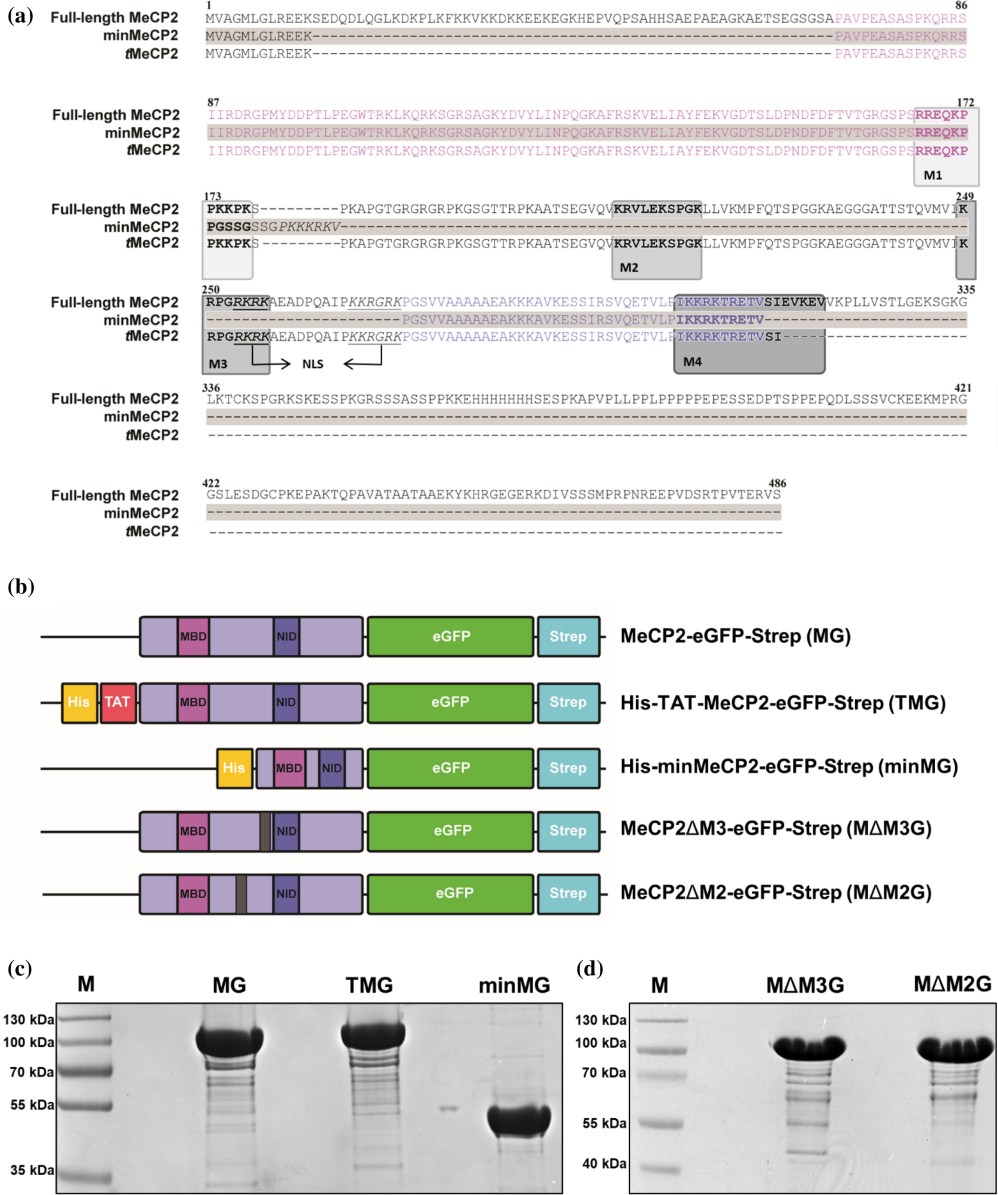
MeCP2 sequence alignment, constructs and SDS–PAGE. (a) Sequence alignment of full‐length MeCP2 (e2 isoform), minMeCP2, and *t*MeCP2. Sequences corresponding to the MBD and NID are colored in light and dark purple, respectively. Sequences that may be responsible for MeCP2 uptake are boxed, the nuclear localization sequence is underlined and marked with arrows. (b) Schematic representation of the MeCP2‐eGFP, TAT‐MeCP2‐eGFP, minMeCP2‐eGFP, MeCP2ΔM3‐eGFP, and MeCP2ΔM2‐eGFP constructs and their appropriate acronyms. (c) SDS–PAGE of MeCP2‐eGFP, TAT‐MeCP2‐eGFP, and minMeCP2‐eGFP, along with the peqGOLD V protein marker. (d) SDS–PAGE of MeCP2ΔM3‐eGFP and MeCP2ΔM2‐eGFP along with the peqGOLD IV protein marker.

The TAT‐MeCP2‐eGFP (TMG), MeCP2‐eGFP (MG), minMeCP2‐eGFP (minMG), MeCP2ΔM3‐eGFP (MΔM3G), and MeCP2ΔM2‐eGFP (MΔM2G) protein constructs (Figure 1), as well as the e1 isoforms of MeCP2 (Me1G) and TAT‐MeCP2 (TMe1G), both also tethered to eGFP (Figure S1) were expressed, purified, and tested for suitability for downstream experiments as previously described (Beribisky et al., 2022). While the theoretical molecular weight of MeCP2 is 53 kDa (and that of its MeCP2‐eGFP counterpart is 81 kDa), owing to MeCP2's polyproline tract, all MeCP2‐eGFP constructs have migrated significantly slower compared to what was to be expected for proteins of their size on the SDS–PAGE (Figures 1b and S1), in line with previous observations (Beribisky et al., 2022; Zachariah et al., 2012). To determine whether their presence results in cytotoxicity, murine NIH3T3 fibroblasts were incubated with 4 μM of each of the aforementioned constructs for 1 h and then subjected to an MTT assay (Figure S2a). There were no changes in NIH3T3 cell viability observed compared to that of cells incubated with MeCP2 storage buffer, indicating that none of the aforementioned protein constructs are toxic to NIH3T3 cells following 1 h of incubation.

### 2.2 MG transduces into NIH3T3 cells and localizes to the nucleus

**FIGURE 2:**
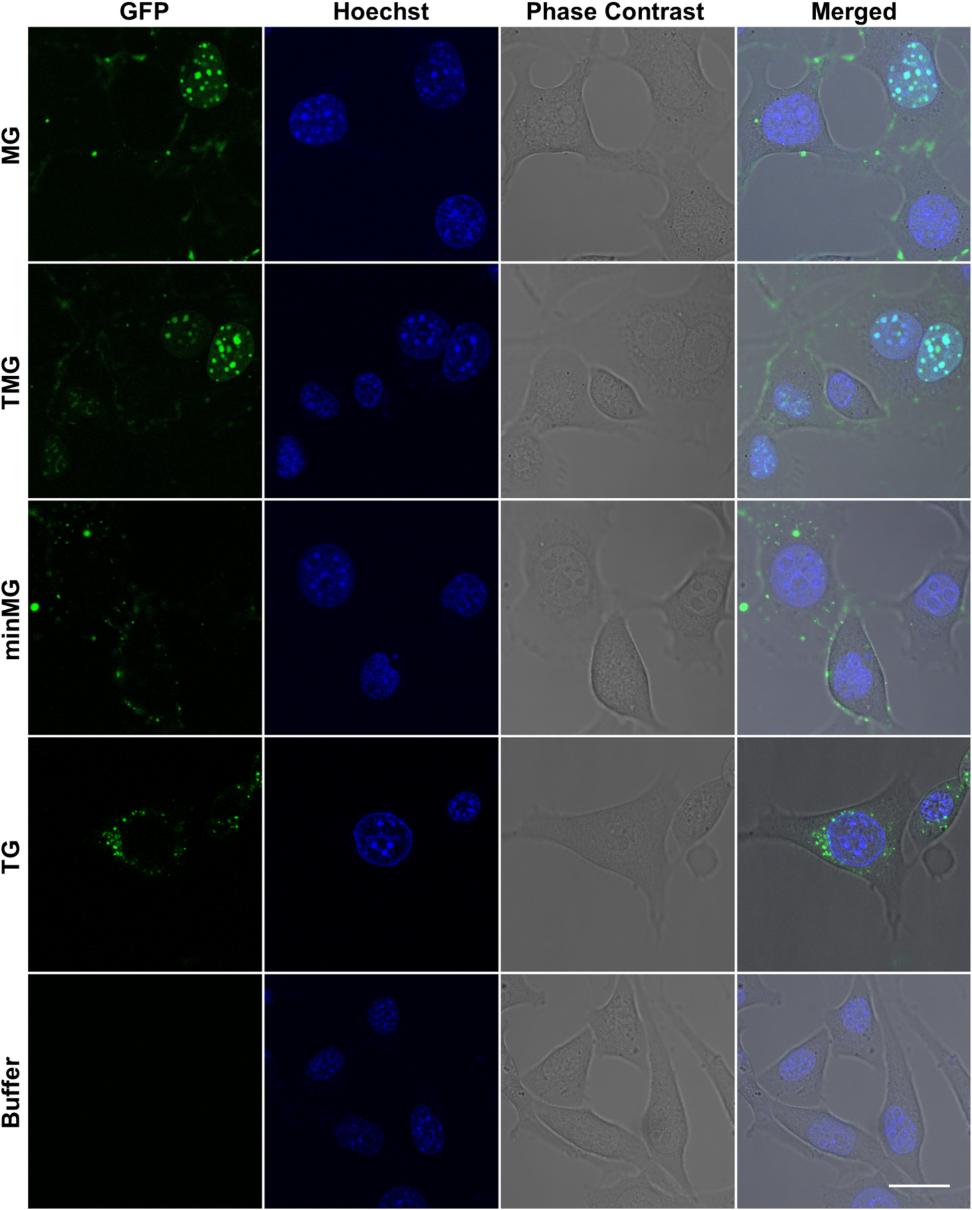
Investigation of MeCP2 uptake. Live‐cell imaging of NIH3T3 cells incubated with 4 μM MG, TMG, minMG, TG, or MeCP2 buffer. Scale bar = 20 μm.

To investigate MeCP2's cellular uptake abilities and to compare them to those of other MeCP2‐derived constructs, the transduction of its GFP‐tethered variants into NIH3T3 cells was studied by live‐cell imaging, the most suitable technique to accurately assess CPP internalization (Beribisky et al., 2022; Richard et al., 2003). The aforementioned cell line was chosen for this study due to MeCP2's ability to localize to its heterochromatic centers in the nuclei (Ito‐Ishida et al., 2020; Li et al., 2020), a property that may help to assess its uptake and subsequent localization. Incubation with 4 μM MG resulted in its accumulation mainly at heterochromatic foci in NIH3T3 nuclei with some additional protein presence in the cytoplasm (Figure 2). These observations are akin to those reported for TMG, a CPP fusion protein (Steinkellner et al., 2022), which is known to transduce across cell membranes and localize to the heterochromatin in the nuclei (Figure 2). The e1 isoform of the Me1G construct was also found to be transduction‐capable (Figure S1c) along with its TM1eG counterpart (Figure S1). Transduced TAT‐eGFP (TG) has exhibited cytoplasmic localization (Figure 2), in line with previous findings (Beribisky et al., 2022; Caron et al., 2001). No apparent morphological differences between protein‐ and buffer‐treated cells (Figure 2) were observed, further supporting the findings that the proteins in question do not exert any toxic effects on NIH3T3 cells at the applied concentrations.

As MeCP2 is an IDP, we asked whether its unstructured portions play a role in the transduction process. To address this question, we performed live‐cell imaging on NIH3T3 cells incubated with 4 μM minMG, a MeCP2 variant that contains only the MBD and NID (Tillotson et al., 2017) and is hence largely devoid of disordered MeCP2 components (Figure 1a). This protein failed to enter the cells, some residual minMG was found to adhere to cell membranes (Figure 2). This lack of minMG uptake strongly suggested that the disordered parts of MeCP2 may be implicated in the transduction process.

**FIGURE 3:**
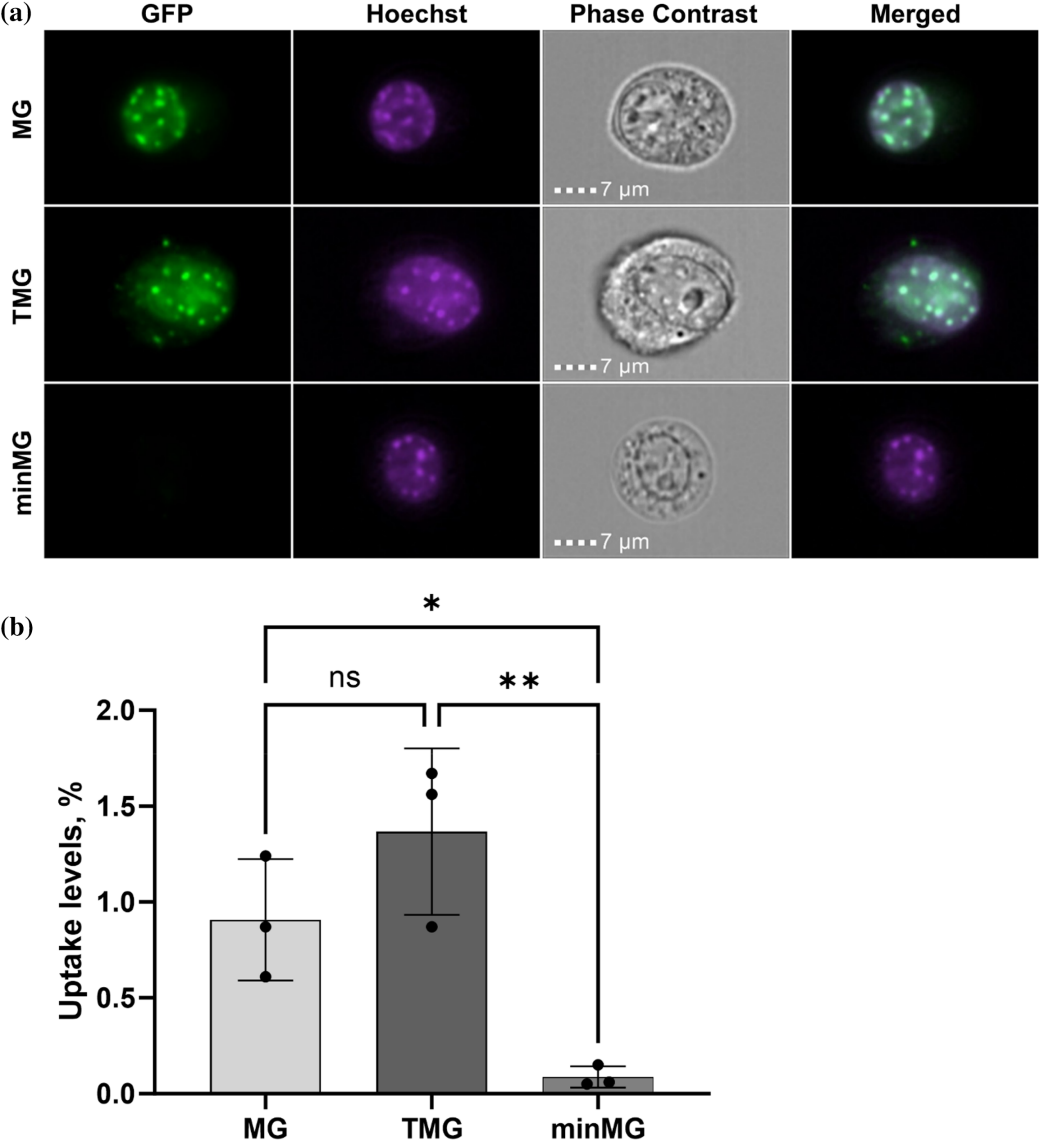
(a) Representative imaging flow cytometry images of NIH3T3 cells incubated with 3 μM MG, TMG, minMG. (b) Quantification of TMG, MG, and minMG (3 μM each) nuclear uptake using imaging flow cytometry. The data represents mean values ± SDs of three biological replicates. ANOVA was used to determine significant differences between protein variant uptake levels. ns, not significant; *p ≤ 0.05; **p ≤ 0.01.

To confirm our findings, we evaluated and quantified the transduction abilities of MG, TMG, and minMG by imaging flow cytometry, as the combination of cell sorting and fluorescence microscopy can serve as an adequate tool for assessing CPP uptake. Similar to the live‐cell imaging data, cells were considered positive for the protein of interest when the fusion protein fluorescence signal was co‐localized with heterochromatic foci in the nucleus (Figure S3). Both MG‐ and TMG‐positive NIH3T3 cells were detected, with strong nuclear heterochromatic localization (Figure 3a). There were 1.5 times more TMG‐positive nuclei than MG‐positive nuclei, while almost no cells with heterochromatic protein co‐localization were detected with minMG (Figure 3b). Taken together, this data further supports our live‐cell imaging findings that MG can transduce across cell membranes and localize to the nuclei in a manner similar to that of TMG and that the disordered parts of MeCP2 may facilitate this process.

### 2.3 Endosomes are implicated in MG internalization

**FIGURE 4:**
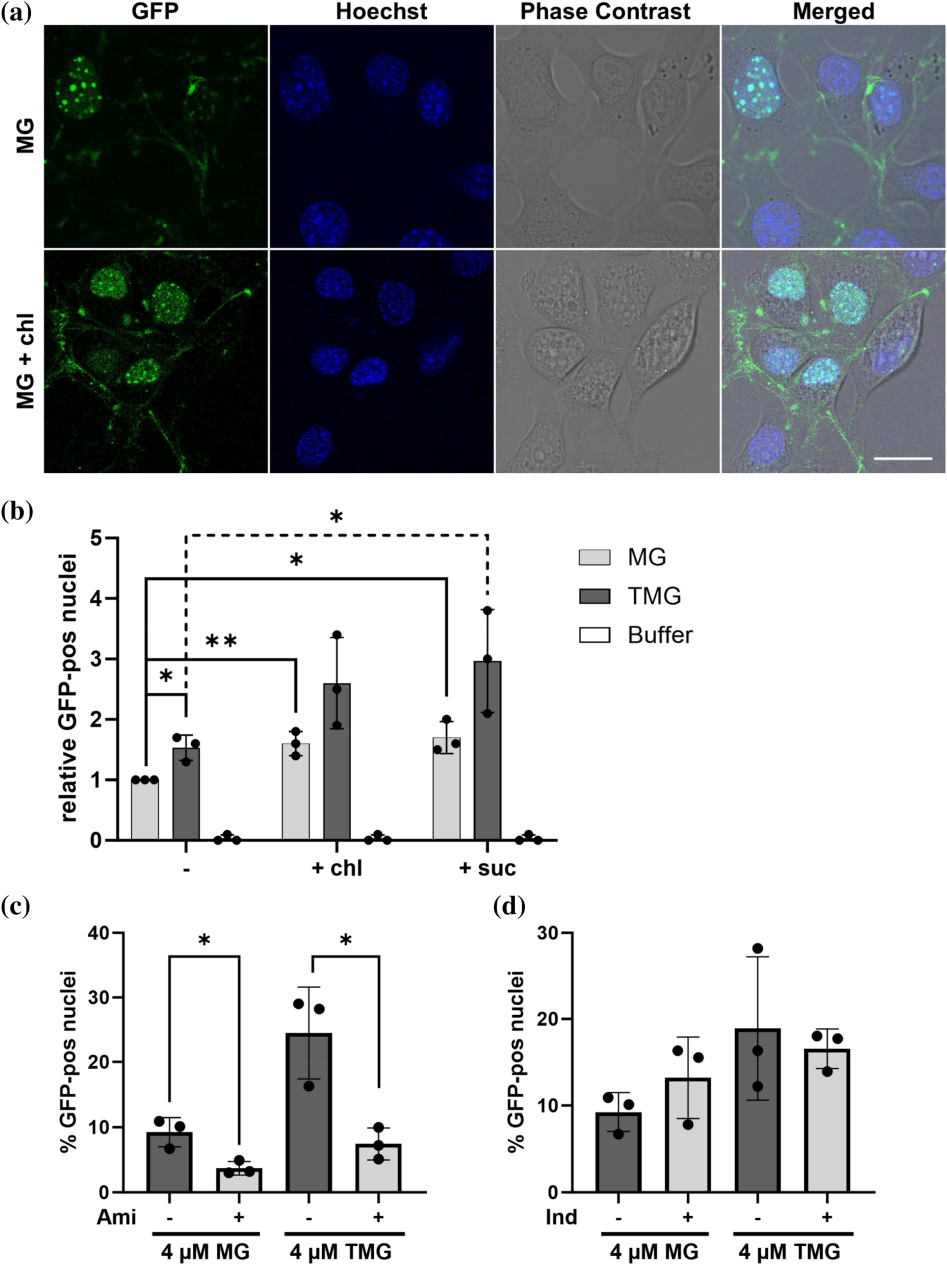
Endosomal involvement in MG and TMG uptake. (a) Live‐cell imaging of NIH3T3 cells incubated with 4 μM MG (top) and 4 μM MG co‐incubated with 100 μM chloroquine (bottom). Scale bar = 20 μm. (b) Quantification of cellular uptake in NIH3T3 cells treated with 4 μM MG and 4 μM TMG and buffer‐treated cells (dark gray) alone and in combination with 100 μM chloroquine (+ chl) or 80 mM sucrose (+ suc). The data are presented as the means ± SDs of three biological replicates. Student's *t* test was used to determine the significance of the differences between MG and TMG uptake upon the addition of endosome‐disrupting compounds; *p ≤ 0.05, **p ≤ 0.01. (c) Quantification of uptake of 4 μM MG and 4 μM TMG alone or in combination with 0.5 mM amiloride into NIH3T3 cells. (d) Quantification of uptake of 4 μM MG and 4 μM TMG alone or in combination with 0.5 mM indomethacin into NIH3T3 cells. Student's *t* test was used to determine the significance of the differences between MG and TMG uptake upon the addition of endocytosis inhibitors; *p ≤ 0.05, **p ≤ 0.01.

Endosomes are known to be involved in the uptake of various CPPs (Caron et al., 2004; LeCher et al., 2017; Sahni et al., 2020). To determine whether MG also samples the endosomal pathway during internalization, NIH3T3 cells were co‐incubated with MG and endosome‐disrupting compounds (Figure 4). Chloroquine and sucrose are compounds that are known to destabilize endosomes and, as such, promote the clearance and escape of their contents (Capasso et al., 2009; Caron et al., 2004). We reasoned that if MG is indeed trafficked via endosomes, their disruption should lead to an increase in protein uptake and nuclear localization. Indeed, when subjected to 100 μM chloroquine, MG transduction was significantly enhanced, and the number of MeCP2‐positive nuclei increased (Figure 4a).

To quantify the increase in MG uptake resulting from the addition of 100 μM chloroquine or 80 mM sucrose and compare it to the transduction efficiency of TMG, the number of MG‐ and TMG‐positive cells in the absence or presence of these compounds was quantified using the ImageXpress® Pico imaging system and CellReporterXpress software (Figure 4b). A cell was considered positive when the nuclear stain co‐localized with increased GFP fluorescence compared to that of a buffer control. For data analysis, the results were normalized to the MG uptake levels. Upon the addition of chloroquine, a 60% increase in nuclear localization occurred with MG, while the addition of sucrose resulted in even greater (70% increase) protein incorporation. These observations clearly point to the utilization of the endosomal pathway during the MG transduction process. A more marked increase in transduction levels occurred for TMG, demonstrating a stronger effect of endosome disrupting compounds on TAT‐mediated uptake.

To obtain additional insight into the specific endosomal pathways sampled by either MG or TMG, co‐incubation of these two proteins with inhibitors of specific endocytosis pathways was performed. Amiloride and indomethacin are two compounds which are known to target two CPP‐sampled endocytosis pathways: Macropinocytosis (Gump et al., 2010; Yang et al., 2018) and caveolae‐mediated endocytosis (Yang et al., 2018; Yumoto et al., 2012) respectively. MG uptake levels were significantly reduced by the addition of amiloride (Figure 4c) and remained unchanged at the presence of indomethacin (Figure 4d). TMG transduction on the other hand, was also found to be affected by amiloride (Figure 4c) as well as to be trend wise sensitive to indomethacin (Figure 4d). These observations suggest that both MG and TMG sample macropinocytosis during the uptake process, while TMG may also use caveolae‐mediated endocytosis as an additional mode of entry.

Upon direct comparison, TMG was shown to enter NIH3T3 cells 50% more efficiently compared to MG (Figure 4b), which is generally in agreement with our imaging flow cytometry data (Figure 3b). The addition of chloroquine and sucrose resulted in an observable increase in the difference between the TMG and MG uptake efficiencies (63% for chloroquine and 74% for sucrose). These findings clearly demonstrate the utility of TAT as a CPP, as its presence significantly enhanced MeCP2 transduction.

We have also utilized the ImageXpress® Pico imaging system and CellReporterXpress software to compare uptake levels of both MeCP2 isoforms. Me1G has displayed comparable transduction levels to its e2 counterpart (Figure S1). As in the case of MeCP2e2, the presence of TAT enhanced MeCP2e1 uptake, as TMe1G transduced more efficiently compared to Me1G. Coupled with our live‐cell imaging experiments, these findings clearly indicate that both MeCP2 isoforms display significant cell internalization capabilities.

### 2.4 MeCP2 uptake sequence mapping

We then proceeded to perform a more thorough sequence analysis of MeCP2 to determine whether a specific portion of this protein is responsible for its cellular internalization. The MeCP2 sequence was analyzed using CPPSite 2.0 (Agrawal et al., 2016), a software that scans protein sequences for putative CPP motifs. The top four sequences (termed M1–M4) were characterized by a high abundance of basic amino acids as well as by the presence of proline residues. They also showed partial alignment to known CPPs (Table 1, highlighted in Figure 1a). M1 and M2 display sequence homology to CPP motifs derived from the respiratory syncytial virus (RSV) (Langedijk et al., 2004), while M4 possesses a degree of similarity to a CPP derived from protein C from the Dengue virus (DENV) (Freire et al., 2014; Zhang et al., 2021). Remarkably, M3 bears a striking resemblance to TAT, a well‐described CPP used in multiple protein replacement therapy studies (Gong et al., 2006; Trazzi et al., 2018; Vyas et al., 2012).

**TABLE 1:** In silico prediction of CPP motifs in MeCP2 using CPPSite 2.0.

Motif name	M1	M2
MeCP2 sequence	** 168 REQKPPKKPK 177 **	** 210 KRVLEKSPGK 219 **
Alignment consensus	R‐‐‐PPKKPK	KR+‐‐K‐PGK
CPP motif sequence	** RSKNPPKKPK **	** KRIPNKKPGK **
CPP type	RSV‐B3	RSV‐A7
Motif name	M3	M4
MeCP2 sequence	** 249 ‐‐‐‐KRPGRKR 255 **	** 303 IKKRKTRETVSIEV‐‐‐‐‐KEV 319 **
Alignment consensus	‐‐‐‐KRPXR+R	IKKXK‐‐‐‐‐‐IXV‐‐‐‐‐KE+
CPP motif sequence	** YGRKKRPQRRR **	** IKKSKA‐‐‐‐‐INVLRGFRKEI **
CPP type	TAT	pep^R^

*Note*: The MeCP2 sequences are marked in purple and the corresponding CPP sequences are marked in red. In the alignment consensus sequence, a “+” denotes an amino acid residue of a similar type, and “X” indicates differing residues.

**FIGURE 5:**
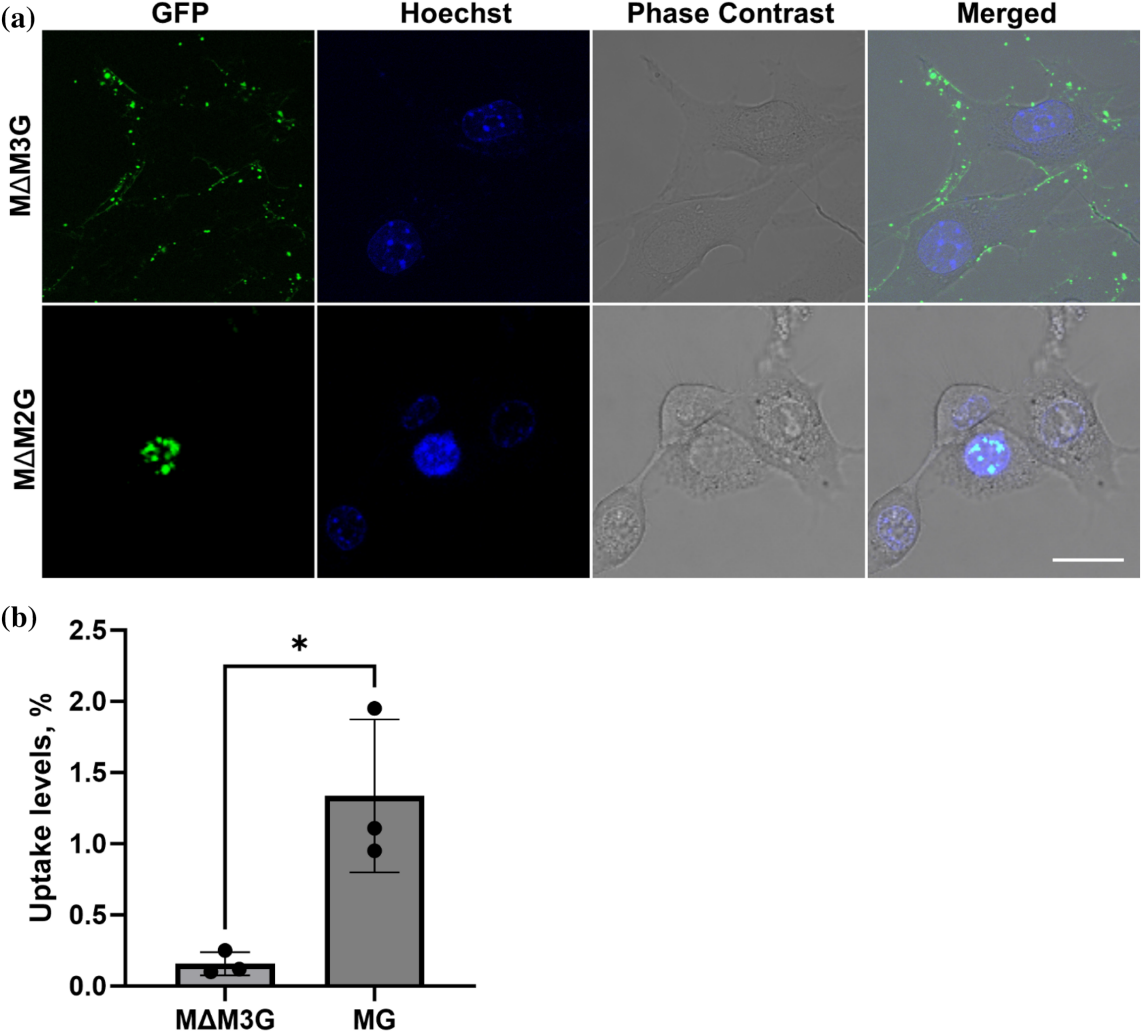
Live‐cell imaging of NIH3T3 cells incubated with putative MeCP2 constructs lacking the M3 or the M2 sequence. (a) Live‐cell imaging of NIH3T3 cells incubated with 3 μM MΔM3G (top), MΔM2G (bottom). Scale bar = 20 μm. (b) Comparison of MG and MΔM3G uptake (3 μM each) using imaging flow cytometry. The data represents mean values ± SDs of three biological replicates. ANOVA was used to determine significant differences between protein variant uptake levels; ns, not significant; *p ≤ 0.05.

The sequences M1 and M4 were excluded as those are present in the transduction‐incompetent minMeCP2, making them dispensable for MeCP2 uptake. A small portion of M4 is not present in minMeCP2; however, it only harbors one basic amino acid residue (Figure 1a), making it a very poor CPP candidate. On the other hand, M2 and M3 are absent in minMeCP2 but present in another shortened MeCP2 protein construct termed *t*MeCP2 that retains transduction capability (Zhang, Cattoglio, et al., 2023). Protein constructs containing M2 or M3 tethered to eGFP on either their N‐ or C‐terminus (Figure S4a) were expressed, purified (Figure S4b) and were all found not to affect cell viability in an MTT assay (Figure S2b). Their ability to mediate cellular uptake was then assessed by live‐cell imaging with NIH3T3 cells. No internalization of either M2‐eGFP or eGFP‐M2 was detected (Figure S4c). However, eGFP‐M3 was found to be transduction‐capable, with trace amounts of this protein found in the cytoplasm (Figure S4d), suggesting that M3 may play a role in MeCP2 incorporation. To verify this observation, MΔM3G, a MG protein construct lacking the M3 motif (Figure 1b) was expressed, purified (Figure 1d) and subjected to live‐cell imaging with NIH3T3 cells. This construct failed to transduce (Figure 5a), while MΔM2G, lacking the M2 motif, on the other hand, retained its incorporation capability (Figure 5a). In addition, almost no MΔM3G NIH3T3 cells in imaging flow cytometry were observed (Figure 5b). These findings clearly demonstrated that the M3 sequence is necessary for MeCP2 uptake.

### 2.5 Internalized MeCP2 recruits its putative association partner HDAC3

**FIGURE 6:**
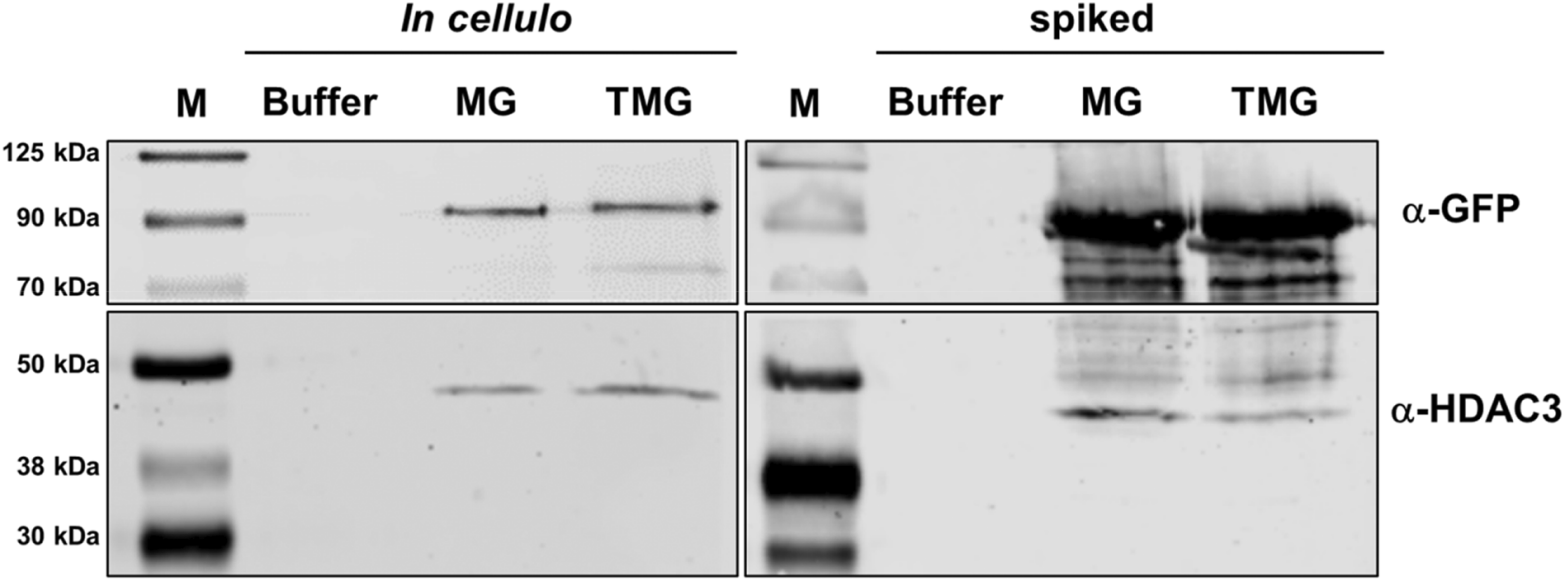
CoIP of MG and TMG. MG and TMG (top panels) both recruit HDAC3 (bottom panels) following transduction into intact cells (left panels, protein concentrations employed are 1.5 μM for each construct) as well as when added directly to the cell lysate (right panels, protein concentrations employed are 150 nM for each construct). M—Chameleon DUO pre‐stained marker.

We then proceeded to test the ability of the transduced MG to recruit known interaction partners. One such partner is HDAC3, which associates with MeCP2 through the histone deacetylase complex (Nott et al., 2016). Internalized MG and TMG have shown a comparable ability to recruit HDAC3 (Figures 6 and S5a), consistent with previous findings where *t*MeCP2 tethered to either TAT or the mini‐protein ZF5.3 were used (Zhang, Cattoglio, et al., 2023). MG and TMG were also shown to successfully interact with HDAC3 when they were added directly to NIH3T3 lysates (Figures 6 and S5b). All nuclear fractions were found to be free of cytoplasmic contamination (Figure S5c). These findings clearly show that recombinant MG also reaches the cell nuclei in sufficient amounts to be functional in cellulo.

## 3 DISCUSSION

The protein MeCP2 plays a prominent role in the regulation of gene expression. Aberrant levels of this protein have been linked to RTT, a highly debilitating, currently incurable neurodevelopmental disease (Lyst & Bird, 2015), and other disorders (Geerdink et al., 2002; Katz et al., 2016). One prospective RTT therapy option involves administering exogenous MeCP2 tethered to the extensively studied CPP TAT (Mi et al., 2000; Ruseska & Zimmer, 2020; Schwarze & Dowdy, 2000), thereby replenishing functional MeCP2 levels in the CNS. This approach has exhibited significant potential in treating RTT (Beribisky et al., 2022; Steinkellner et al., 2022; Zhang, Cattoglio, et al., 2023) and other neurological disorders (Gong et al., 2006; Trazzi et al., 2018; Vyas et al., 2012).

MeCP2 possesses two structured domains, MBD and TRD, which are responsible for protein–DNA (Wakefield et al., 1999) and protein–protein (Forlani et al., 2010; Kruusvee et al., 2017; Tillotson et al., 2017) interactions respectively, but is otherwise largely disordered. Some structural (Beribisky et al., 2022) as well as functional significance has been ascribed to the IDP portions of MeCP2 (Buschdorf & Stratling, 2004; Ortega‐Alarcon et al., 2021); however, the properties and functional capabilities of these regions warrant additional investigation.

SPs are a class of proteins characterized by an abnormally high charge compared to that of other members of the proteome (Cronican et al., 2010). SPs can be artificially generated (Lawrence et al., 2007; Thompson et al., 2012); however, naturally occurring SPs that vary in function from growth factors to transcription factors as well as viral capsid proteins (Cronican et al., 2011; Zhang et al., 2021) have also been described. In general, proteins are considered SPs when they possess a high overall charge and a CM_W_ greater than one at physiological pH, with 0.75 being considered the cut‐off for a protein that can be transduction‐capable (Ma et al., 2020; Thompson et al., 2012). MeCP2 has a CM_W_ of 0.73 and a very high theoretical net charge of +38.5 at pH 7.2. Its charge is slightly higher than that of +36 GFP, an artificially generated SP GFP derivative (Lawrence et al., 2007). In addition, both values exceed those of endonuclease 8‐like 1 (CM_W_ = 0.51, charge of +23.7 at pH = 7.2), a component of the DNA base excision repair machinery (Hazra et al., 2002), which has demonstrated the ability to transduce non‐covalently bound cargo (Cronican et al., 2011). Taken together, this data suggests that the presence of many disordered portions coupled with some of its physicochemical properties, such as an abnormally high charge and CM_W_, make MeCP2 a potential SP candidate.

When studying CPP or protein cellular uptake, several considerations are of importance. First, cell fixation must be avoided as this technique generates abnormal CPP localization patterns (Richard et al., 2003) in part due to endosomal rupture (Teo et al., 2021). Hence, especially when evaluating initial MG transduction capabilities, live‐cell imaging was employed as the method of choice, as in previous work (Cronican et al., 2010; Richard et al., 2003). In addition, non‐incorporated membrane‐bound CPP fusion protein or SP may complicate analysis. To remove membrane‐adhering protein, the glycosaminoglycan heparin, a competitive inhibitor of TAT (Hakansson et al., 2001) that has been used previously on multiple occasions (Console et al., 2003; Flinterman et al., 2009), was also employed here.

MeCP2 is known to co‐localize to heterochromatic foci (Ito‐Ishida et al., 2020) in the nuclei of numerous cell types (including NIH3T3, which are used in this work), and has also been linked to phase transitions of heterochromatic condensates, which, as with other proteins (Kim et al., 2013; Mackenzie et al., 2017), carries clinical implications (Li et al., 2020). This property can be exploited to evaluate the uptake ability of the MG protein construct.

In addition to live‐cell imaging, we employed imaging flow cytometry to study MeCP2 internalization. While traditional flow cytometry methods were previously used on numerous occasions for this purpose (Cronican et al., 2011; Illien et al., 2016; Zhang, Cattoglio, et al., 2023), this technique suffers from several drawbacks. Residual membrane‐adhering protein can overestimate uptake levels (Richard et al., 2003) which also hinders the accurate assessment of nuclear localization. Cell nuclei with an incorporated CPP fusion protein can be isolated and studied (Zhang, Cattoglio, et al., 2023); however, the isolation process may introduce additional inaccuracies from residual membrane‐adhering protein. Imaging flow cytometry provides not only population frequencies (% internalized) but also offers insight into protein localization in individual cells (Zhang, Kanoatov, et al., 2023). Consequently, intact live cells can be used to accurately assess the levels of nuclear CPP or SP uptake.

Both the live‐cell imaging (Figure 2) and imaging flow cytometry data (Figure 3) clearly demonstrated that MG successfully transduces across the membranes of NIH3T3 cells and compartmentalizes to their nuclei, with visible accumulation at heterochromatic centres in a fashion similar to that of its TMG counterpart. No such uptake was observed in the case of minMG, a construct largely devoid of MeCP2's IDP components. These findings point to the involvement of the disordered parts of this protein in the transduction process. Other SPs were also shown to harbor disproportionately high amounts of IDP portions (Cronican et al., 2011; Ma et al., 2020; Thompson et al., 2012), which may also contribute to their cell‐penetrating abilities.

The nuclear localization levels of both MG and TMG, as measured by imaging flow cytometry, were on the order of 1% (Figure 3b). While TAT fusion protein transduction efficiency can vary greatly and largely depends on the protein cargo (Albarran et al., 2005; Chugh & Eudes, 2008), studies on a shortened MeCP2 construct termed *t*MeCP2 have quantified its nuclear levels as a result of TAT‐mediated uptake at 20% or higher (Zhang, Cattoglio, et al., 2023). Several factors account for this difference. First, it is likely that the transduction levels of the full‐length MeCP2 constructs employed here (MG and TMG) would be lower than those of their *t*MeCP2 counterparts. This would be in line with the finding that full‐length TMG exhibits inferior uptake levels compared to TAT‐minMeCP2‐eGFP (Beribisky et al., 2022), another truncated MeCP2 construct with high sequence similarity to *t*MeCP2 (Figure 1a). In addition, different study methods were employed. The aforementioned analysis of *t*MeCP2 uptake was performed using fluorescence correlation spectroscopy, while in our case imaging flow cytometry was the method of choice.

While our work here has focused on the e2 isoform of MeCP2, another isoform of this protein, e1, also exists. This isoform was shown to be more prominently implicated in RTT onset (Djuric et al., 2015; Zachariah et al., 2012). The e1 and e2 sequences exhibit a high degree of sequence similarity with the exception of the presence of a polyalanine tract at the e1 isoform's N‐terminal end (Djuric et al., 2015). We have found that MeCP2e1 can also readily transduce and localize to heterochromatic foci of NIH3T3 cells at levels comparable to those of its e2 counterpart (Figure S1d). Any potentially slight difference in uptake may be explained by MeCP2e1's lower CM_W_ compared to MeCPe2 (0.67 vs. 0.73). Nevertheless, these findings clearly indicate that the e1 isoform of MeCP2 also possesses appreciable cell transduction capabilities.

Endosomal involvement in CPP fusion protein uptake has been clearly established with TAT‐fusion proteins largely employing either macropinocytosis or caveolae‐mediated endocytosis (Ruseska & Zimmer, 2020; Zhang, Cattoglio, et al., 2023). Furthermore, *t*MeCP2 was recently shown to utilize two components of the endosomal pathway during its cellular incorporation: C core vacuole/endosome tethering (CORVET) and homotypic fusion and protein sorting (HOPS) complexes, which are responsible for early and late endosomal trafficking, respectively (Zhang, Cattoglio, et al., 2023). TAT‐*t*MeCP2, on the other hand, was shown to be transported independently of both CORVET and HOPS, indicating that TAT may sample other endosomal as well as non‐endosomal pathways (Zhang, Cattoglio, et al., 2023). To determine whether endosomes also play a role in the uptake of full‐length MG and compare it to the endosomal involvement in TMG incorporation, NIH3T3 cells were co‐incubated with the aforementioned proteins and either chloroquine or sucrose, both known endosome‐disrupting compounds (Capasso et al., 2009; Caron et al., 2004). Clear increases in the number of MG‐positive cells were observed upon the addition of chloroquine or sucrose (60% and 70%, respectively). These increases were even more pronounced for TMG (Figure 4b). Chloroquine is known to inhibit late endosome and lysosome acidification, which in turn prompts their rupture (Varkouhi et al., 2011), while sucrose accumulates in endosomes, causing them to swell (Ciftci & Levy, 2001). Given these facts, it is conceivable that late endosomes play a tangible role in MG and TMG trafficking.

In addition, the endocytosis pathway utilized by both MG and TMG was probed through the use of endocytosis inhibitors. Amiloride and indomethacin are two such inhibitors, which interfere with macropinocytosis or caveolae‐mediated endocytosis, by inhibiting Na^+^/H^+^ exchangers (Gump et al., 2010; Koivusalo et al., 2010; Yang et al., 2018) and targeting folate receptors (Gandek et al., 2023; Yang et al., 2018; Yumoto et al., 2012) respectively. MG uptake levels experienced a clear decrease upon the addition of amiloride (Figure 4c), while remaining unaltered at the presence of indomethacin (Figure 4d). TMG transduction on the other hand was sensitive to amiloride addition (Figure 4c) and has also shown a downward trend upon indomethacin co‐incubation (Figure 4d). These observations may indicate that endocytotic MG uptake is largely governed by macropinocytosis, while TMG gains cellular entry by macropinocytosis and possibly also by caveolae‐mediated endocytosis. These observations are in line with previous findings which point to macropinocytosis being the dominant endocytotic CPP uptake mechanism (Ruseska & Zimmer, 2020). However, the endosomal pathways employed by various CPPs in general as well as *t*MeCP2 and full‐length MeCP2 in particular may vary due to differences in their protein sequences.

Based on previous (Cronican et al., 2011; Ma et al., 2020) and current findings, SP sequences that mediate cellular uptake, are most likely to be positively charged and disordered, that is possess CPP‐like properties. By utilizing CPPSite 2.0 (Agrawal et al., 2016), a CPP sequence search algorithm, four putative sequences in MeCP2 with the highest scores were obtained. All four sequences were found to be rich in arginine and lysine and all but one contained proline. These three residues are present in multiple CPP classes and are known to play an integral role in their transduction activity (Ruseska & Zimmer, 2020; Sadler et al., 2002). All sequences displayed a homology to CPP motifs derived from various viral proteins from RSV, HIV‐1, and DENV (Table 1). Based on a combination of computational and experimental work, it was determined that the CPP culprit sequence in MeCP2 is a basic stretch between the MBD and the TRD that bears high resemblance to TAT (Figures 5 and S4). While MeCP2's structured domains, the MBD and NID are indeed crucial for protein function (Tillotson et al., 2017), no high‐scoring CPP motifs were detected within the majority of their sequences (Figure 1a), which would have pointed to their implication in MeCP2 cell entry.

The TAT uptake mechanism, while not entirely deciphered, is known to involve endocytosis at lower concentrations and direct translocation at higher CPP levels (Ruseska & Zimmer, 2020). Notably, the role of the arginine residues in TAT is crucial for both uptake modes. In the case of endocytosis, the side chains of these residues are known to interact with heparin sulfates on the cell membrane and also bring about actin rearrangement (Christianson & Belting, 2014; Futaki et al., 2007). When direct translocation occurs, these arginine residues are thought to recognize the phosphate portions of the membrane, which prompts subsequent hydrophobic interactions between the aliphatic portions of the CPP and the bilayer, inducing pore formation triggering TAT fusion protein entry (Herce et al., 2009).

Upon direct comparison, TMG was found to transduce more readily than its MG counterpart, in both our imaging flow cytometry (Figure 3b) and the ImageXpress® Pico (Figure 4b) experiments. Such a difference in incorporation exists due to largely four reasons. First, it has been suggested that a minimum of eight positive charges are necessary for efficient uptake to be initiated (Ruseska & Zimmer, 2020; Wang et al., 2014). While the TAT peptide harbors eight such charges, the M3 motif in MeCP2 has only six, due to the lack of two basic amino acid residues (lysine and arginine, Table 1) in its sequence. Second, the presence of aromatic residues in CPP motifs was shown to facilitate intercalation of the CPP into the cell membrane (Jobin et al., 2015; Madani et al., 2011; Yu et al., 2018) and enhance endosomal escape (Jobin et al., 2015; Lonn et al., 2016). The complete TAT peptide contains a tyrosine residue at its N‐terminal end, while no such residue is present in M3. The M3 flanking sequences in MeCP2 may also inhibit this motif's transduction capability. Finally, as TMG but not MG transduction has shown to be affected by a caveolae‐mediated endocytosis inhibitor, it is conceivable that the complete TAT sequence allows the former construct to sample the caveolae‐mediated endocytosis pathway, which may not be accessible to MG. These factors explain the diminished uptake ability of M3 in MeCP2 compared to the full‐length TAT in TMG.

On the other hand, it is also possible that other positively charged portions of MeCP2 act in concert with M3 to facilitate the cellular incorporation of the protein, as local patches of positive charge, which can be supplied by MeCP2's flexible, disordered portions are a required prerequisite for initiating protein transduction pathways (Ruseska & Zimmer, 2020). However, the presence of M3 seems indispensable for any appreciable MeCP2 uptake to take place. It is also possible that the M3 sequence in the TMG protein construct plays a role in enhancing its uptake by acting as a second pseudo‐TAT motif.

The M3 sequence also harbors part of MeCP2's bipartite nuclear localization signal (NLS) (Nan et al., 1996). This kind of NLS sequence (underlined and marked with arrows in Figure 1a) is found in multiple proteins, with positively charged residues being critical for nuclear transport (Lu et al., 2021). However, in the case of MeCP2, the NLS was found to be redundant for nuclear localization (Lyst et al., 2018), indicating that its nuclear import sequence requirements are likely less stringent than those of CPP or SP transduction. Nevertheless, the presence of at least part of the NLS in the pseudo‐TAT MeCP2 sequence may point to another CPP utility—enhancing nuclear localization.

Finally, we asked whether MeCP2 can be transduced in amounts appreciable enough to impart its function as a transcriptional regulator. One of these functions is engaging in protein–protein interactions with other transcriptional modulators, such as HDAC3, TBLR1 and Sin3A (Kruusvee et al., 2017; Nan et al., 1998; Nott et al., 2016). Indeed, following uptake into NIH3T3 cells, both MG and TMG successfully recruited HDAC3 (Figure 6), in line with previous findings in which TAT‐*t*MeCP2 was found to sequester this protein upon internalization (Zhang, Cattoglio, et al., 2023). These findings further confirm MG's transduction capability as well as its functionality upon subsequent nuclear localization.

This study has a number of limitations. A more thorough investigation of the effect of MeCP2's sequences, including its structured domains, on the protein's uptake may provide additional insight into the more intricate aspects of MeCP2 internalization. In addition, murine fibroblasts which are not phenotypically relevant to RTT were employed in this work, largely due to MeCP2's ability to localize to their heterochromatic foci (Ito‐Ishida et al., 2020; Li et al., 2020). TAT‐MeCP2‐eGFP constructs have also been showed to be transduction‐capable in MeCP2‐deficient cells previously with subsequent nuclear compartmentalization (Beribisky et al., 2022).

In summary, this work clearly demonstrated that MeCP2 possesses significant cell‐penetrating properties akin to those of numerous SPs, with subsequent compartmentalization to cell nuclei. The internalization process is enhanced by endosome‐disrupting compounds, clearly indicating that MeCP2 uptake utilizes the endosomal pathway, in particular, macropinocytosis. Remarkably, a stretch in MeCP2's IDP portion which bears a striking resemblance to TAT is necessary for MeCP2 transduction. As this sequence lacks a number of the basic and aromatic amino acids present in the full‐length TAT, MG internalization occurs at a lower efficiency than that of TMG. Like its TMG counterpart, transduced MG readily recruits the known MeCP2 binding partner HDAC3. These findings provide novel insights into the structure and function of SPs in general and MeCP2 in particular and may prove beneficial for the utilization of protein chemistry in protein‐based drug design.

## 4 MATERIALS AND METHODS

### 4.1 Expression and purification of the MECP2 and eGFP constructs

DNA fragments encoding MG, TMG (both e1 and e2 isoforms), minMG, MΔM3G, MΔM2G, M2‐eGFP, eGFP‐M2, M3‐eGFP, eGFP‐M3, and eGFP were each cloned into the plasmid pET‐28a. The sequence encoding the Strep affinity tag flanked the protein‐coding portions on the 3′ end (Figure 1a for the e2 isoforms and Figure S1 for the e1 isoforms). The MeCP2 constructs were expressed as described elsewhere (Beribisky et al., 2022; Steinkellner et al., 2022), M2‐eGFP, eGFP‐M2, M3‐eGFP, and eGFP‐M3 were expressed in lysogeny broth medium with protein expression induced at OD_600_ of 0.8 with 1 mM isopropyl β‐D‐1‐thiogalactopyranoside with subsequent overnight incubation shaking at 240 rpm at 20°C.

The procedure by which MeCP2‐eGFP, TAT‐MeCP2‐GFP (both e1 and e2 isoforms), minMG, MΔM3G, and MΔM2G were purified has been outlined previously (Beribisky et al., 2022; Steinkellner et al., 2022). Briefly, the constructs were captured from *E. coli* lysate using Strep‐Tag affinity chromatography (IBA, #2‐5014‐001) with gel filtration chromatography used as a second purification step. Following three rounds of lipopolysaccharide (LPS) removal, the samples were flash‐frozen and stored in DPBS, 200 mM NaCl, 10% glycerol, 0.05% CHAPS, pH = 7.2 at −80°C. All samples were analyzed using 12% SDS–PAGE, the PeqGOLD V (Figure 1c) or PeqGOLD IV (Figure 1d) protein marker (VWR, #27‐2210 and VWR, #27‐2110, respectively) was used for molecular weight estimation.

M2‐eGFP, eGFP‐M2, M3‐eGFP, eGFP‐M3, and eGFP were purified in a similar fashion to their MeCP2 counterparts, with two notable differences. The Strep‐column‐bound protein was treated with a wash buffer containing 500 mM NaCl. Following Strep‐column chromatography, the samples were buffer‐exchanged into DPBS, 10% glycerol, pH = 7.2 (eGFP‐Buffer). After elution from the gel filtration column, the proteins were simply concentrated to a final volume of 1 mL using a 10,000 MWCO concentrator, aliquoted, flash‐frozen and stored at −80°C. LPS extraction was not performed. The samples were analyzed using 12% SDS–PAGE, and Precision Plus Protein Dual Xtra Pre‐stained protein standards (Bio‐Rad, #1610377) were used for molecular weight estimation.

### 4.2 Cell culture

NIH3T3 murine embryonic fibroblasts were cultured in Dulbecco's modified Eagle's medium (DMEM, Gibco, #41966) supplemented with 10% fetal bovine serum (FBS, Sigma, #F9665) and a 1% penicillin–streptomycin mixture (Gibco, #15140122) at 37°C under a humidified atmosphere of 5% CO_2_.

### 4.3 Cell viability assays

The 3‐(4,5‐dimethylthiazol‐2‐yl)‐2,5‐diphenyltetrazolium bromide (MTT) assay was performed as previously described (McKenzie et al., 2019) with slight modifications. NIH3T3 cells were seeded at 2 × 10^4^ cells per well in a 96‐well plate (Greiner, #655180). The next day, the cells were treated with various protein constructs at 4 μM or 10 μM for 1 or 18 h corresponding to their use in live‐cell imaging experiments. The medium was then removed, and the cells were incubated with 110 μL of medium containing 455 μg/mL MTT for 2 h. Then, 110 μL of extraction buffer (20% sodium dodecyl sulfate and 50% N,N‐dimethylformamide, pH = 4.7) was added to each well, followed by a final incubation overnight at 37°C. The absorbance at 570 nm was measured using a BioTek plate reader. Cell viability is represented as a percentage of the untreated cells.

### 4.4 Live‐cell imaging

A total of 25,000 murine NIH3T3 cells per well were seeded on 8‐well collagen IV‐treated μ‐slides (Ibidi, 80822) 1 day prior to the experiments. On the day of the experiment, the medium was removed, and protein variants were diluted in culture medium to the indicated concentrations and applied directly to the cells for 1 h. For live‐cell imaging experiments testing endosomal involvement in protein uptake, the cells were co‐incubated with 100 μM chloroquine. Following incubation, 1.5 μM Hoechst 33342 (Thermo Scientific, #62249) was added for 5 min to counterstain the nuclei. The cells were then washed with DPBS and then three times with 0.5 mg/mL heparin in DPBS to remove external membrane‐bound protein followed by two additional washing steps with DPBS. Subsequently, the cells were incubated with 300 μL of live‐cell imaging solution (Thermo Fisher Scientific, #A14291DJ) and immediately subjected to live‐cell imaging under a confocal microscope (Leica TCS SP8) equipped with temperature and gas controls (37°C, 5% CO_2_).

### 4.5 Imaging flow cytometry

NIH3T3 cells were seeded a day in advance in 6‐well plates (500,000 cells per well) and grown to 80%–90% confluence. MG, TMG, minMG, and MΔM3G (3 μM each) were added to the cells, which were subsequently incubated for 1 h at 37°C. Five minutes prior to the end of the incubation, 1 μM Hoechst 33342 (Thermo Scientific) was added to stain the nuclei. The cell layer was then washed twice with DPBS at room temperature. The membrane‐adhering proteins were removed with 0.5% mg/mL heparin. To detach the cells, 0.05% (v/v) trypsin/EDTA was added, detachment was verified under a light microscope. The cells were collected in 750 μL of medium and centrifuged at 300 × *g* for 5 min at room temperature, after which the supernatant was discarded. The resulting cell pellets were washed twice with 600 μL of ice‐cold DPBS and subsequently centrifuged at 500 × *g* for 5 min at 4°C. The pellets were re‐suspended in 70 μL of ice‐cold DPBS and stored on ice.

An ImageStream®X Mark II imaging flow cytometer (Cytek, Amnis) at the Core Facility Flow Cytometry of the Medical University of Vienna was employed for data acquisition. The following parameters were used: Bright field, fluorescence excitation with a 488 nm laser for GFP detection and with a 405 nm laser for Hoechst detection. Image processing and analysis were performed using IDEAS v6.2 software (Amnis) (Figure S3). Cells were focused with the parameter gradient RMS_BF. Focused cells were then plotted using the parameters AREA_BF against Aspect ratio_BF. Single cells were gated, and as the stop criteria, 5000 singlets were counted. Protein uptake was quantified with the software INSPIRE version 201.1.0.693 (Amnis). Briefly, focused cells and singlets were gated, and the co‐localization wizard in IDEAS software was used to select co‐localized GFP spots and Hoechst‐stained nuclei. The uptake of the MeCP2 constructs into the nucleus was then calculated using the Bright Detail Similarity R3_MC_Ch02_Ch07_Median index. The experiment was performed three times; statistical significance was analyzed through the use of ordinary analysis of variance (ANOVA) and multiple comparisons ANOVA in Prism software.

### 4.6 CPP site mapping

The mapping of putative CPP sites in MeCP2 was performed using CPPSite 2.0 software (Agrawal et al., 2016; Kardani & Bolhassani, 2021). The MeCP2e2 wild‐type sequence was used, and the four top‐scoring results were selected. *t*MeCP2 and minMeCP2 sequences were also employed in the analysis.

### 4.7 ImageXpress® Pico experiments

The day before the experiments, NIH3T3 cells were seeded at a density of 1.3 × 10^4^ cells per well in clear 96‐well dishes (Corning, #353075). Then, the cells were incubated with 4 μM MG, 4 μM TMG or buffer control for 1 h at 37°C. To investigate endosomal involvement, the cells were co‐incubated with either 100 μM chloroquine or 80 mM sucrose. To study endocytosis inhibition, the cells were pre‐incubated with 0.5 mM amiloride or 0.5 mM indomethacin for 1 h at 37°C, followed by a co‐incubation with either one of these compounds with 4 μM MG, 4 μM TMG or buffer control for the same duration and temperature. Following incubation, Hoechst 33342 was added at a final concentration of 3.6 μM for 5 min to counterstain the nuclei. The cells were then washed with DPBS twice and then three times with 0.5 mg/mL heparin in DPBS to remove membrane‐bound proteins. This step was followed by two additional washing steps with DPBS to completely remove the heparin. Finally, 200 μL of growth medium was added to each well, and the cells were immediately imaged using the ImageXpress Pico automated cell imaging system (Molecular Devices). The number of nuclei with Hoechst 33342‐positive areas co‐localized with GFP fluorescence, as well as the total number of nuclei, were analyzed using CellReporterXpress software (Molecular Devices). The number of positive nuclei was normalized to MG alone, with *n* = 3 biological replicates.

### 4.8 Co‐immunoprecipitation of HDAC3 with MG and TMG

An experimental procedure described previously (Zhang, Cattoglio, et al., 2023) was employed with a number of modifications. NIH3T3 cells were seeded in three 10 cm dishes per condition (3.5 × 10^6^ cells/dish/15 mL medium) and subsequently incubated on the next day with 1.5 μM MG, TMG or MeCP2 storage buffer for 1 h at 37°C. Following treatment with 0.5 mg/mL heparin, the cells were collected, and the nuclear lysates were isolated using the REAP method (Suzuki et al., 2010) with minor modifications. The isolates were stored in CoIP buffer (20 mM HEPES, 10 mM KCl, 1.5 mM MgCl_2_, pH = 7.6). The nuclear lysates were disrupted for 90 s using a Covaris sonicator, followed by a 10 min incubation at 300 rpm and 37°C and the addition of NaCl to a final concentration of 430 mM. An additional sonication step using a Covaris sonicator was then performed for 20 s, after which the lysates were incubated for 1 h and shaken with gentle agitation at 4°C. The lysates were then centrifuged for 10 min at 16,000 × *g* and 4°C. β‐Mercaptoethanol (BME) was added to the supernatants to a final concentration of 15 mM, and the samples were further diluted with CoIP buffer to a final NaCl concentration of 150 mM. The protein concentration was determined using the Bio‐Rad protein assay (Bio‐Rad, #5000006). For the CoIP experiment itself, MagStrep “type 3” XT beads (IBA, #2‐4090‐002) were used at a ratio of 0.85 nmol of total protein per 1 μL of Strep beads (20 μL of bead solution). The beads were pre‐washed with CoIP buffer three times before the addition of cell lysates. A minimum of 100 μg of isolate per sample was used. The lysate‐bead mixture was then incubated overnight with gentle rotation at 4°C. The next day, the supernatant was removed, and the beads were washed thrice with CoIP buffer. The protein complexes were eluted from the beads using 20 μL of CoIP buffer supplemented with 1× SDS loading solution and 10% (v/v) BME, with subsequent heating for 3 min at 95°C. Input, flowthrough, and elution samples, along with 5 μL of Chameleon Duo pre‐stained marker (LI‐COR, #928‐60000), were all loaded on a 12% acrylamide gel, which was run at 80 V for 2 h. Western blotting was performed using the iBlot® 2 Gel Transfer Device (Invitrogen, IB21001). Intercept® Blocking Buffer (LI‐COR, #927‐60001) was used to block the membrane for 1 h with gentle rotation at room temperature. Primary staining was then carried out using anti‐GFP (Abcam, #ab290, 1:1000), anti‐HDAC3 (CST, #85057S, 1:1000), and anti‐β‐tubulin (Sigma, #T4026, 1:2000) antibodies in Intercept® Antibody Diluent (LI‐COR, 927‐65001) overnight with rotation at 4°C. The next day, the membranes were incubated with anti‐mouse (LI‐COR, #926‐68070) or anti‐rabbit (LI‐COR, #926‐32211) secondary antibodies diluted 1:20,000 in Intercept® Antibody Diluent for 1 h under gentle agitation at 4°C. The blots were then imaged using Odyssey CLx (LI‐COR Biosciences) and analyzed using ImageStudio software (LI‐COR Biosciences).

For the lysate‐spiking experiments, NIH3T3 nuclear lysates were isolated as described above, and MG or TMG was added to a final concentration of 150 nM. The downstream treatment of the samples was conducted as described above.

## AUTHOR CONTRIBUTIONS

**Alexander V. Beribisky:** Conceptualization; investigation; writing – original draft; methodology; validation; visualization; writing – review and editing; formal analysis; data curation. **Anna Huber:** Investigation; writing – review and editing; visualization; methodology. **Victoria Sarne:** Investigation; methodology; visualization; writing – review and editing; validation. **Andreas Spittler:** Investigation; visualization; resources. **Nyamdelger Sukhbaatar:** Visualization; writing – review and editing; methodology. **Teresa Seipel:** Investigation; visualization; writing – review and editing. **Franco Laccone:** Conceptualization; investigation; funding acquisition; project administration; supervision; writing – review and editing. **Hannes Steinkellner:** Conceptualization; investigation; funding acquisition; methodology; validation; writing – review and editing; visualization; project administration; supervision; formal analysis.

## FUNDING INFORMATION

This research was funded by the Italian Rett Syndrome Association (AIRETT ETS) grant (H. S.). F. L. is inventor of the patent WO2007115578 A8, “Synthetic mecp2 sequence for protein substitution therapy”; holder of the patent: Georg August University, Universitätsmedizin Göttingen.

## CONFLICT OF INTEREST STATEMENT

The authors A. V. B., A. H., V. S., A. S., N. S., and T. S. declare no conflicts of interest.

## Supporting information


**Figure S1:** Isolation and uptake studies of (TAT‐) MeCP2e1‐eGFP constructs. (a) Schematic representation of the TAT‐MeCP2e1‐eGFP, MeCP2e1‐eGFP constructs and their appropriate acronyms. The differing N‐terminal portion of this MeCP2 isoform is denoted in light gray. (b) SDS–PAGE of TAT‐MeCP2e1‐eGFP and MeCP2e1‐eGFP, M—Precision Plus Protein Dual Xtra marker. (c) Live‐cell imaging of NIH3T3 cells incubated with 4 μM MeCP2e1‐eGFP (top) or TAT‐MeCP2e1‐eGFP (bottom). Scale bar = 20 μm. (d) Quantification of cellular uptake in NIH3T3 cells incubated with 4 μM e1 or e2 isoforms of MeCP2‐eGFP, TAT‐MeCP2e1‐eGFP, and buffer‐treated cells. The data are presented as the means ± SDs of three biological replicates.
**Figure S2:** Effects of the investigated constructs on cell viability. NIH3T3 cells were treated with: (a) 4 μM MG, minMG, TMG, or MeΔM3G for 1 h. (b) 10 μM M2‐eGFP, eGFP‐M2, M3‐eGFP, eGFP‐M3, or eGFP for 18 h. Cell survival was determined using the MTT assay. Conditions were normalized to the corresponding buffer control. The data represents the mean values ± SDs of two biological replicates with three technical replicates each.
**Figure S3:** Imaging flow cytometry gating scheme. (a) Cell focusing using the parameter gradient RMS_BF. (b) Plot of focused cells and gated cells; 5000 singlets were counted. (c) Co‐localization GFP positive cells and Hoechst‐stained nuclei.
**Figure S4:** The use of putative CPP‐eGFP motifs to map the MeCP2 uptake sequence. (a) Schematic representation of putative MeCP2 uptake sequence motifs tethered to eGFP at either the N‐ or C‐terminus. (b) SDS–PAGE of purified M2‐eGFP, eGFP‐M2, M3‐eGFP, and eGFP‐M3. M—Precision Plus Protein Dual Xtra marker. (c) Live‐cell imaging of NIH3T3 cells incubated with (c) eGFP‐M2 (top) and M2‐eGFP (bottom), each 10 μM or (d) eGFP‐M3 (top) and M3‐eGFP (bottom), each 10 μM. Scale bar = 20 μm.
**Figure S5:** Western blots of the nuclear fractions of (a) NIH3T3 cells and (b) NIH3T3 cell lysates spiked with MG or TMG and stained for the presence of eGFP‐tethered MeCP2 (α‐GFP antibody) and HDAC3 (α‐HDAC3 antibody). (c) Western blots of the nuclear fractions of NIH3T3 incubated with MG, TMG, and MeCP2 buffer (top) or lysate‐spiked with MG and TMG (bottom), stained for the presence of the cytosolic marker β‐tubulin. M—Chameleon DUO pre‐stained marker. Protein concentrations for the CoIP and spike experiments were 1.5 μM and 150 nM, respectively.
