# Supplementary material for: MeCP2 is a naturally supercharged protein with cell membrane transduction capabilities
Source: Protein Sci. 2024 Sep 14;33(10):e5170. doi: 10.1002/pro.5170 (PMC11400631; doi:10.1002/pro.5170)
Supplement: Supplementary file 1 — Figure S1: Isolation and uptake studies of (TAT‐) MeCP2e1‐eGFP constructs. (a) Schematic representation of the TAT‐MeCP2e1‐eGFP, MeCP2e1‐eGFP constructs and their appropriate acronyms. The differing N‐terminal portion of this MeCP2 isoform is denoted in light gray. (b) SDS–PAGE of TAT‐MeCP2e1‐eGFP and MeCP2e1‐eGFP, M—Precision Plus Protein Dual Xtra marker. (c) Live‐cell imaging of NIH3T3 cells incubated with 4 μM MeCP2e1‐eGFP (top) or TAT‐MeCP2e1‐eGFP (bottom). Scale bar = 20 μm. (d) Quantification of cellular uptake in NIH3T3 cells incubated with 4 μM e1 or e2 isoforms of MeCP2‐eGFP, TAT‐MeCP2e1‐eGFP, and buffer‐treated cells. The data are presented as the means ± SDs of three biological replicates. Figure S2: Effects of the investigated constructs on cell viability. NIH3T3 cells were treated with: (a) 4 μM MG, minMG, TMG, or MeΔM3G for 1 h. (b) 10 μM M2‐eGFP, eGFP‐M2, M3‐eGFP, eGFP‐M3, or eGFP for 18 h. Cell survival was determined using the MTT assay. Conditions were normalized to the corresponding buffer control. The data represents the mean values ± SDs of two biological replicates with three technical replicates each. Figure S3: Imaging flow cytometry gating scheme. (a) Cell focusing using the parameter gradient RMS_BF. (b) Plot of focused cells and gated cells; 5000 singlets were counted. (c) Co‐localization GFP positive cells and Hoechst‐stained nuclei. Figure S4: The use of putative CPP‐eGFP motifs to map the MeCP2 uptake sequence. (a) Schematic representation of putative MeCP2 uptake sequence motifs tethered to eGFP at either the N‐ or C‐terminus. (b) SDS–PAGE of purified M2‐eGFP, eGFP‐M2, M3‐eGFP, and eGFP‐M3. M—Precision Plus Protein Dual Xtra marker. (c) Live‐cell imaging of NIH3T3 cells incubated with (c) eGFP‐M2 (top) and M2‐eGFP (bottom), each 10 μM or (d) eGFP‐M3 (top) and M3‐eGFP (bottom), each 10 μM. Scale bar = 20 μm. Figure S5: Western blots of the nuclear fractions of (a) NIH3T3 cells and (b) NIH3T3 cell lysates spiked with MG or TMG and stain [file PRO-33-e5170-s001.docx]

**Supplementary Information**


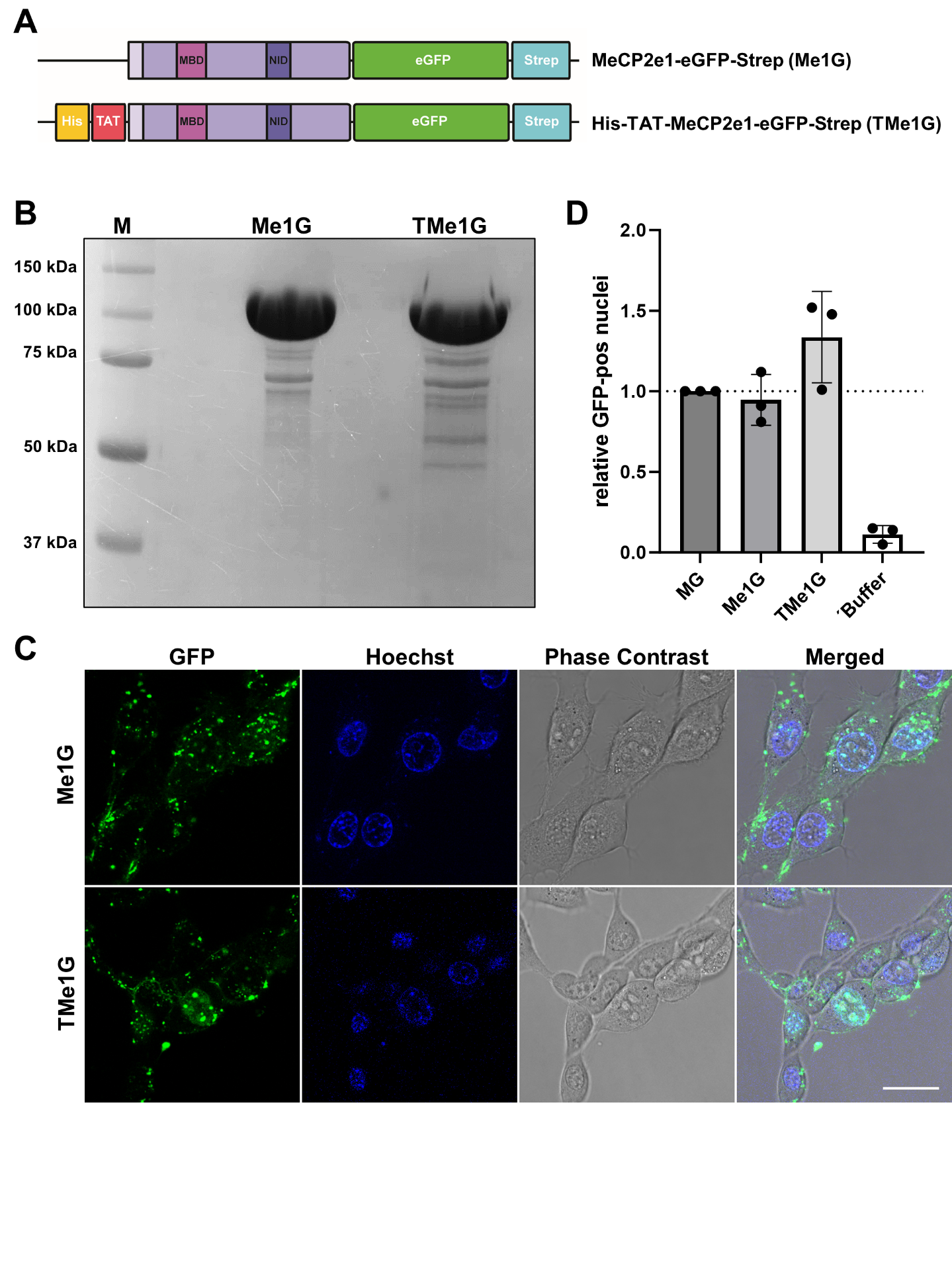


**Figure S1 – Isolation and uptake studies of (TAT-) MeCP2e1-eGFP constructs.** **A** Schematic representation of the TAT-MeCP2e1-eGFP, MeCP2e1-eGFP constructs and their appropriate acronyms. The differing N-terminal portion of this MeCP2 isoform in denoted in light grey. **B** SDS-PAGE of TAT-MeCP2e1-eGFP and MeCP2e1-eGFP, M – Precision Plus Protein Dual Xtra marker. **C** Live-cell imaging of NIH3T3 cells incubated with 4 μM MeCP2e1-eGFP (top) or TAT-MeCP2e1-eGFP (bottom). Scale bar = 20 μm. **D** Quantification of cellular uptake in NIH3T3 cells incubated with 4 μM e1 or e2 isoforms of MeCP2-eGFP, TAT-MeCP2e1-eGFP and buffer-treated cells. The data are presented as the means ± SDs of three biological replicates.


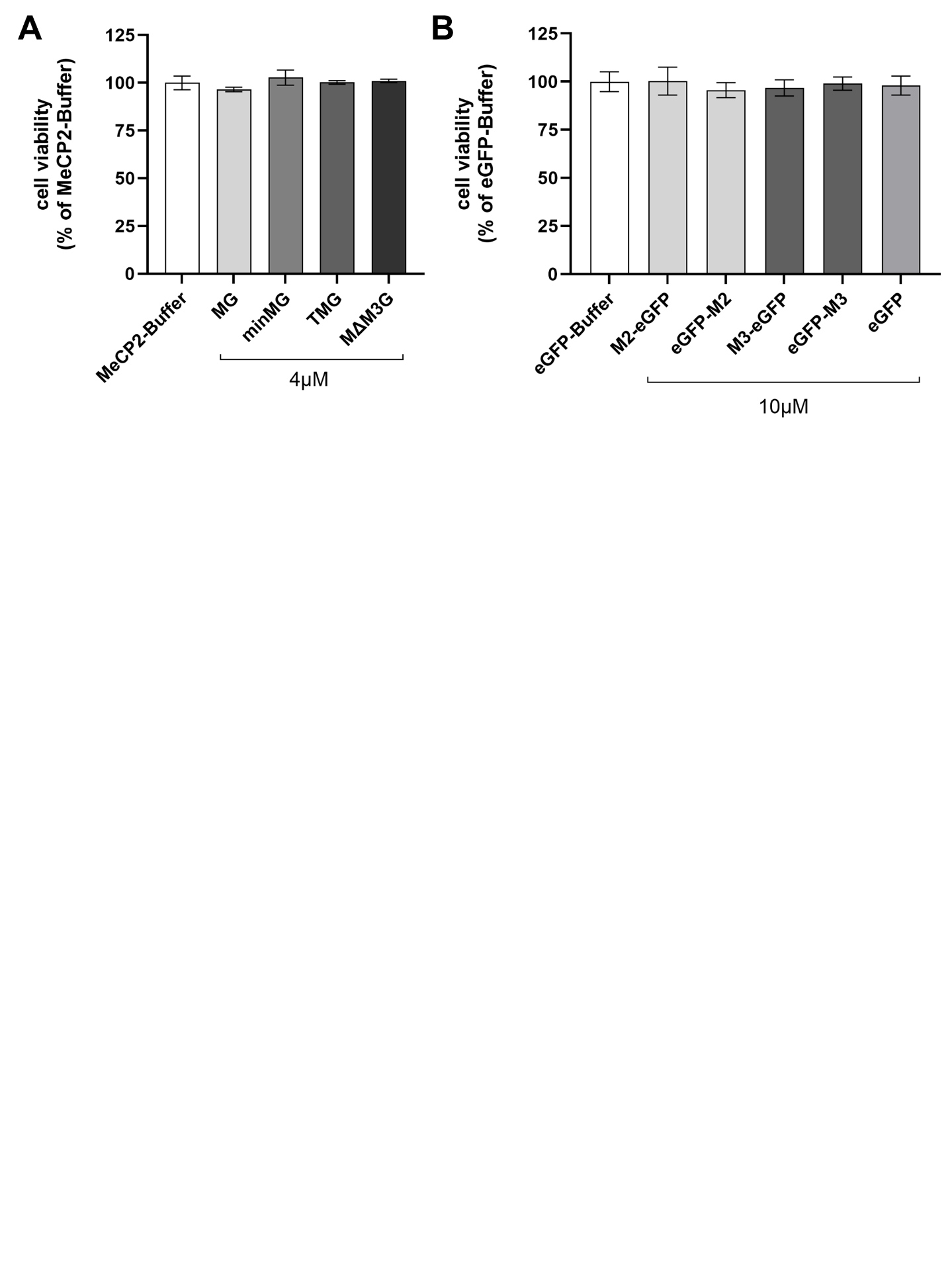


**Figure S2** – **Effects of the investigated constructs on cell viability.** NIH3T3 cells were treated with **A** 4 µM MG, minMG, TMG or MeΔM3G for 1 hour. **B** 10 µM M2-eGFP, eGFP-M2, M3-eGFP, eGFP-M3 or eGFP for 18 hours. Cell survival was determined using the MTT assay. Conditions were normalized to the corresponding buffer control. The data represents the mean values ± SDs of two biological replicates with three technical replicates each.


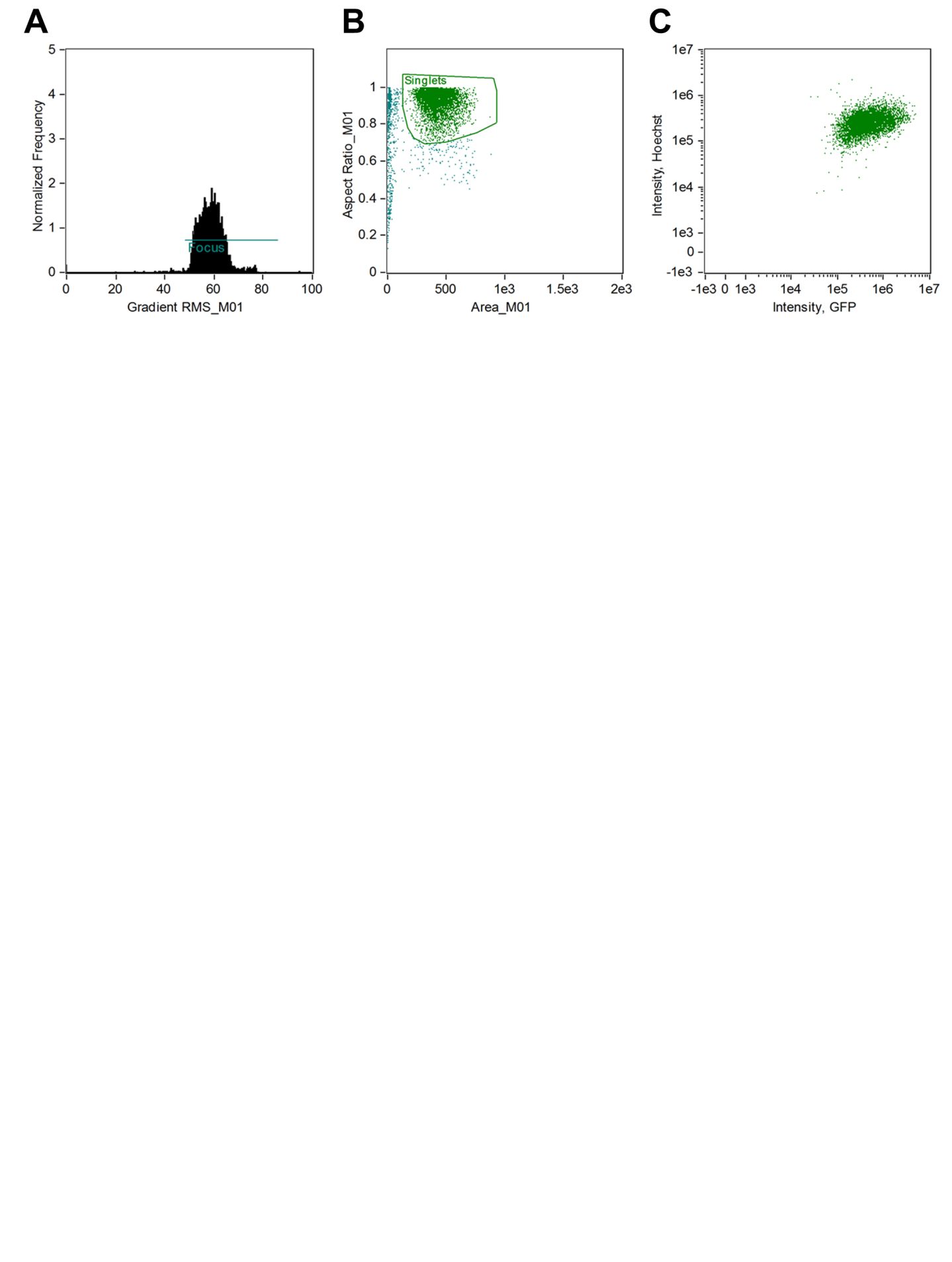


**Figure S3** – **Imaging flow cytometry gating scheme.** **A** Cell focusing using the parameter gradient RMS_BF. **B** Plot of focused cells and gated cells; 5,000 singlets were counted. **C** Co-localization GFP positive cells and Hoechst-stained nuclei.

**
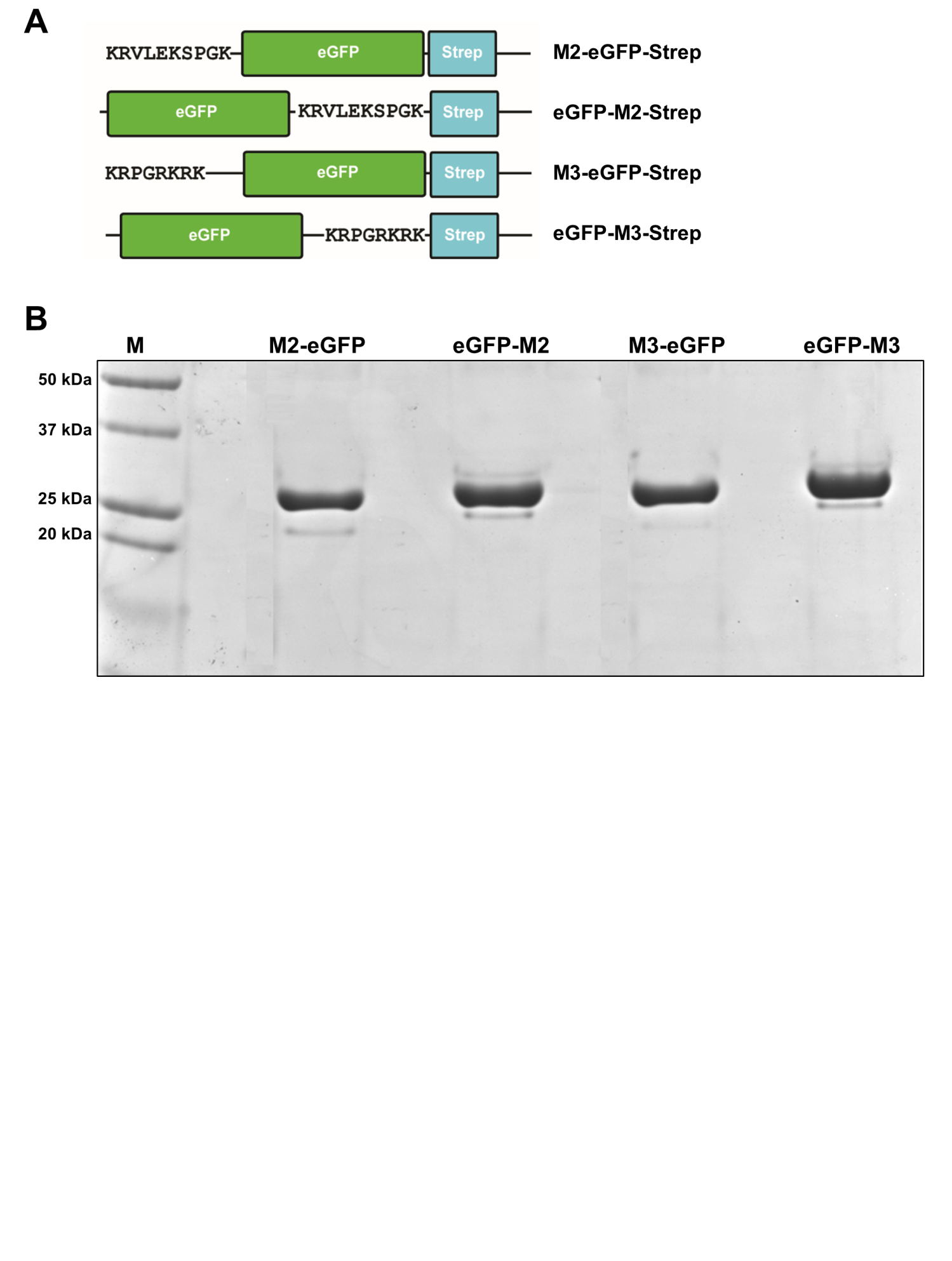
**

**
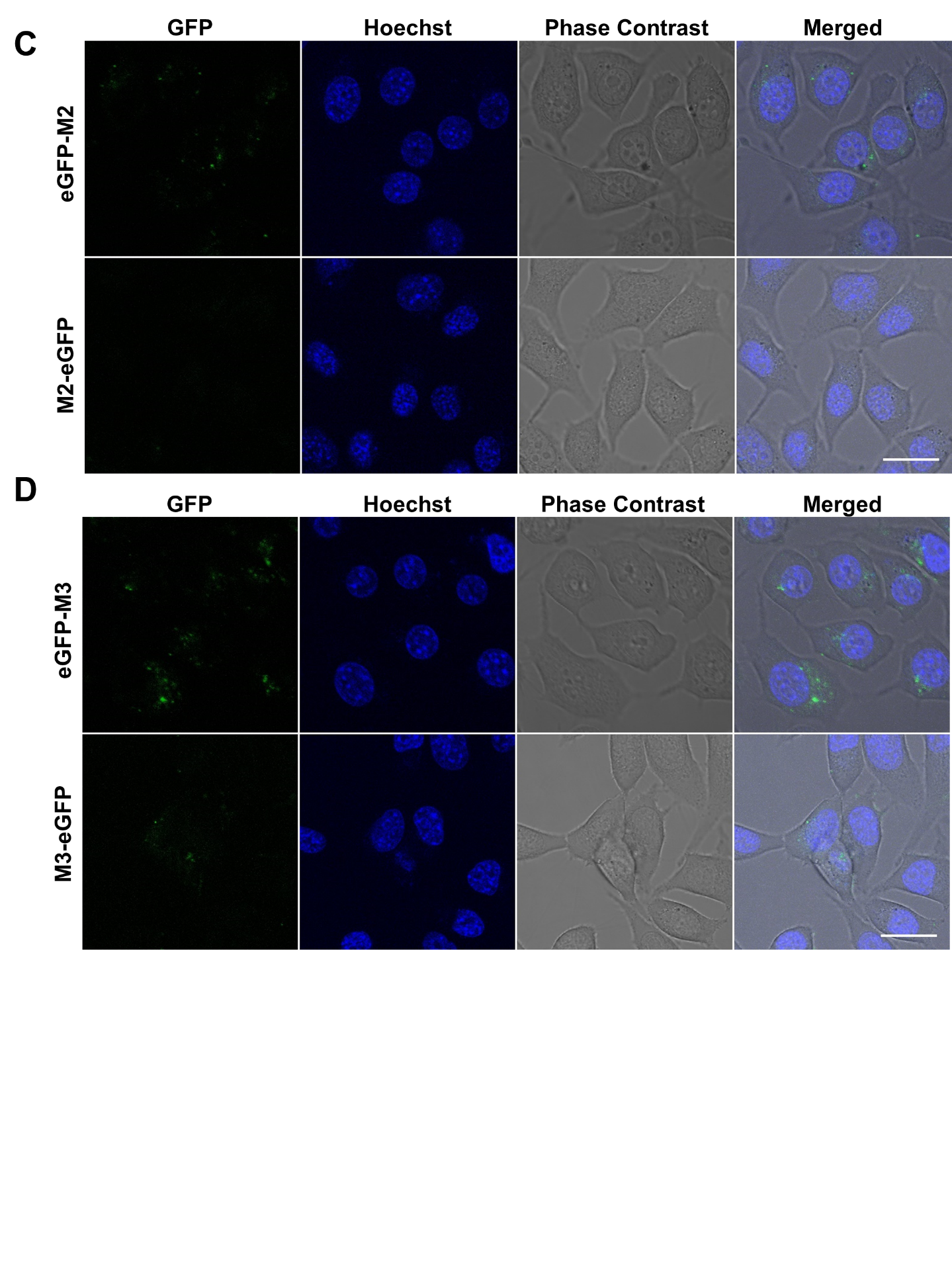
**

**Figure S4** – **The use of putative CPP-eGFP motifs to map the MeCP2 uptake sequence.** **A** Schematic representation of putative MeCP2 uptake sequence motifs tethered to eGFP at either the N- or C-terminus. **B** SDS‒PAGE of purified M2-eGFP, eGFP-M2, M3-eGFP and eGFP-M3. M – Precision Plus Protein Dual Xtra marker. **C** Live-cell imaging of NIH3T3 cells incubated with **C** eGFP-M2 (top) and M2-eGFP (bottom), each 10 µM or **D** eGFP-M3 (top) and M3-eGFP (bottom), each 10 µM. Scale bar = 20 μm.


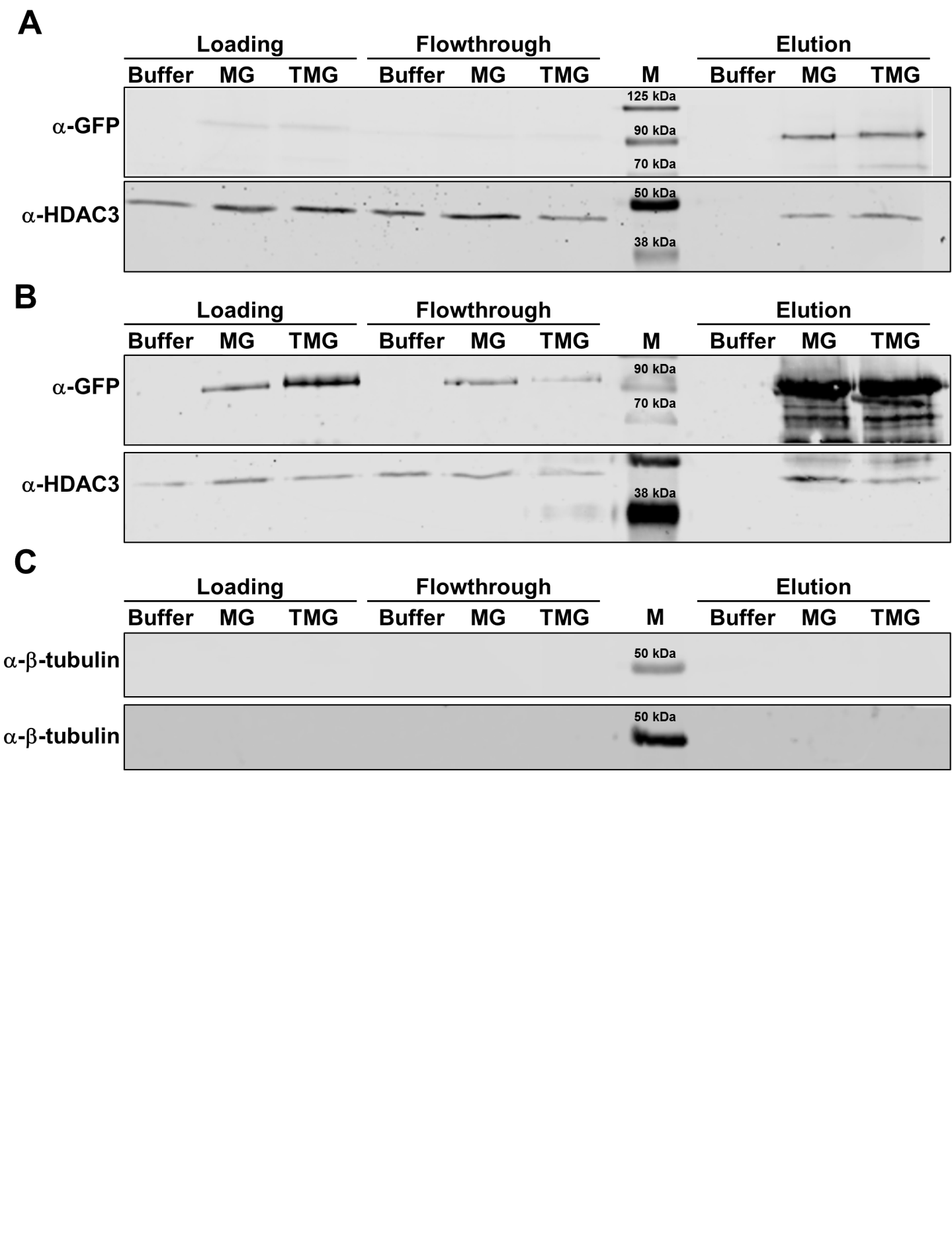


**Figure S5** – Western blots of the nuclear fractions of **A** NIH3T3 cells and **B** NIH3T3 cell lysates spiked with MG or TMG and stained for the presence of eGFP-tethered MeCP2 (α-GFP antibody) and HDAC3 (α-HDAC3 antibody). **C** Western blots of the nuclear fractions of NIH3T3 incubated with MG, TMG and MeCP2 buffer (top) or lysate-spiked with MG and TMG (bottom), stained for the presence of the cytosolic marker β-tubulin. M – Chameleon DUO pre-stained marker. Protein concentrations for the CoIP and spike experiments were 1.5 μM and 150 nM respectively.
